# New Reassortant H5N6 Highly Pathogenic Avian Influenza Viruses in Southern China, 2014

**DOI:** 10.3389/fmicb.2016.00754

**Published:** 2016-05-19

**Authors:** Peirong Jiao, Jin Cui, Yafen Song, Hui Song, Zhishan Zhao, Siyu Wu, Nannan Qu, Nianchen Wang, Guowen Ouyang, Ming Liao

**Affiliations:** ^1^National and Regional Joint Engineering Laboratory for Medicament of Zoonosis Prevention and ControlGuangzhou, China; ^2^Key Laboratory of Animal Vaccine Development, Ministry of AgricultureGuangzhou, China; ^3^Key Laboratory of Zoonosis Prevention and Control of GuangdongGuangzhou, China; ^4^College of Veterinary Medicine, South China Agricultural UniversityGuangzhou, China; ^5^College of Life Science, South China Agricultural UniversityGuangzhou, China

**Keywords:** H5N6, highly pathogenic avian influenza virus, ducks, evolution, pathogenicity

## Abstract

New reassortant H5N6 highly pathogenic avian influenza viruses (AIVs) were isolated from apparently healthy domestic ducks in Southern China in 2014. Our results show that the viruses grew efficiently in eggs and replicated systemically in chickens. They were completely lethal in chicken (100% mortality), and the mean death time was 6 to 7 days post-inoculation. The viruses could transmit in chickens by naïve contact. BLAST analysis revealed that their HA gene was most closely related to A/wild duck/Shangdong/628/2011 (H5N1), and their NA genes were most closely related to A/swine/Guangdong/K6/2010 (H6N6). The other genes had the highest identity with A/wild duck/Fujian/1/2011(H5N1). The results of phylogenetic analysis showed that their HA genes clustered into clade 2.3.4.4 of the H5N1 viruses and all genes derived from H5 were Mix-like or H6-like viruses. Thus, the new H5N6 viruses were reassortmented of H5N1 and H6N6 virus. Therefore, the circulation of the new H5N6 AIVs may become a threat to poultry and human health.

## Introduction

Waterfowl are a natural reservoir of avian influenza A viruses (Kawaoka et al., 1988). Most subtypes of avian influenza A viruses have been found in aquatic birds (Webster et al., 1992). Southern China is one of the main areas for farming of domestic waterfowl where they often share water with wild aquatic birds. Most avian influenza viruses (AIVs) do not cause disease in humans. However, some are zoonotic meaning that they can infect humans and cause disease. The most well-known example is the avian influenza subtype H5N1 viruses that are currently circulating in poultry in parts of Asia and northeast Africa. These have caused human disease and deaths since 1997. In recent years, a variety of novel avian viruses have been detected in poultry in Guangdong Province. An increasing number of overwhelming viral infections have aroused public awareness of zoonotic avian influenza transmission. Notably, Zhejiang, Hubei, and Guangdong had H5N6 avian influenza in farms in 2014 followed by Hunan and Jiangsu in 2015 (World Organization for Animal Health, 2015). Whenever AIVs are circulating in poultry, sporadic infections and small clusters of human cases are possible in people exposed to infected poultry or contaminated environments. Therefore, new H5N6 viruses should be monitored for their impact on animal and human health.

## Materials and Methods

### Viruses

The H5N6 HPAI viruses A/duck/Guangzhou/018/2014 (018) and A/duck/Guangzhou/021/2014 (021) were isolated from swabs of ducks in a live bird market in Guangdong, China by the Key Laboratory of Animal Disease Control and Prevention, Ministry of Agriculture of P.R. China. They were purified and propagated in 9- to 10-day-old specific-pathogen-free (SPF) embryonated hen eggs. Values of 50% egg infective doses (EID_50_) were calculated by the Reed–Muench method. All experiments were carried out in ABSL-3 facilities.

### Sequence Analysis

The viral genes used in this study were sequenced. Viral RNA was extracted from allantoic fluid with Trizol LS Reagent (Life Technologies, Inc.) and transcribed into cDNA with SuperScript III reverse transcriptase (Invitrogen). PCR amplification was performed using fragment-specific primers that matched the conserved end sequence of the RNA fragments of the influenza virus. The PCR products were purified with the QIAquick PCR purification kit (QIAGEN) and sequenced using an automatic ABI Prism 3730 genetic analyzer (Applied Biosystems) according to the manufacturer’s instructions. The DNA sequences were compiled and edited using Lasergene 7.1 (DNASTAR). A phylogenetic tree of the H5N6 influenza A viruse s was generated by the distance-based neighbor-joining method using software MEGA 5 (Sinauer Associates, Inc., Sunderland, MA, USA). The reliability of the tree was assessed by bootstrap analysis with 1000 replicates. Horizontal distances are proportional to genetic distance. The nucleotide sequences obtained in the present study are available from GenBank under the accession numbers (KX094400-KX094415).

### Infection Study

Six-week-old specific pathogen-free White Leghorn chickens (*n* = 20) were housed in isolator cages and divided into two groups with 10 per group. In each group, seven chickens were intranasally (i.n.) inoculated (10^6^EID_50_ in 0.2 ml) with one of the two viruses. Three chickens were inoculated with 0.2 ml phosphate buffered saline (PBS) as negative controls. All chickens were observed for clinical symptoms for 14 days. Post-infection day 3 (DPI), three inoculated chickens in each group were euthanized to test for viral replication in the organs including the lung, kidney, brain, heart, spleen, and liver. All tissues were collected and titrated for viral infectivity in eggs. All experiments were carried out in ABSL-3 facilities in compliance with the biosafety committee of South China Agriculture University protocols. The chicken handling was performed in accordance with experimental animal administration and the ethics committee of South China Agriculture University guidelines.

## Results

In early March 2014, H5N6 AIVs—A/duck/Guangzhou/018/ 2014 (018) and A/duck/Guangzhou/021/2014 (021)—were isolated from samples of ducks as described (Sun et al., 2011). The viruses grew efficiently in eggs, and both had virus titers >8 log 50% egg infectious dose (EID_50_)/0.1 mL. To understand the pathogenicity of the viruses in chicken, 6-week-old SPF chickens were inoculated intranasally (i.n.) with 10^6^ EID_50_. All chickens were observed for 14 days after infection.

In this test, the infected chickens exhibited anorexia and depression at 1 DPI. All chickens of the 018 group died within 6 DPI. All chickens inoculated with 021 died within 7 DPI. Moreover, the viruses could transmit to chickens by naïve contact. All naïve contact chickens housed with those inoculated with 018 virus died at 4–7 DPI. Three naïve contact chickens died in the 021 group at 5–6 DPI. The viruses replicated systemically in chickens, and this could be detected in all the tested organs by 3 DPI including the heart, liver, spleen, lung, kidney, and brain (**Table 1**). The 018 virus replicated more highly in the lungs (6.75 log_10_ EID_50_). The mean virus titers in the heart, liver, spleen, kidney, and brain were 4.00, 5.92, 5.25, 6.58, and 5.25 log_10_ EID_50_, respectively. The 021 virus replicated in all tested organs and the highest virus titers was detected in the chicken lung (6.17 log_10_ EID_50_; **Table 1**).

**Table 1:** Replication and lethality in chickens of the H5N6 viruses after intranasal infection^a^.

Strains	Titer (log_10_ EID_50_)	Virus replication on 3 days post-inoculation (DPI; log_10_ EID_50_/0.1 ml)^b^ in						Manifestations of chickens
		Heart	Liver	Spleen	Lung	Kidney	Brain	No. D/S/total^c^
018	8.63	4.00 ± 0.00	5.92 ± 0.51	5.25 ± 0.29	6.75 ± 0.87	6.58 ± 0.68	5.25 ± 0.13	10/10/10
021	8.54	3.92 ± 0.01	5.5 ± 0.67	5.08 ± 0.26	6.17 ± 0.35	5.92 ± 0.51	5.33 ± 0.22	10/10/10

*^a^Six-week-old SPF chickens were inoculated intranasally (i.n.) with 10^6^ EID_50_ of 018 virus or 10^6^ EID_50_ of 021 virus in a volume of 100 μl, respectively; three chickens in each group were euthanized on 3 DPI, and virus titers were measured in samples of heart, liver, spleen, lung, kidney, and brain in the chicken embryo. ^b^For statistical analysis, a value of 1.5 was assigned if the virus was not detected from the undiluted sample in three embryonated hen eggs. Virus titers were expressed as means standard deviation in log_10_ EID_50_/0.1 ml of tissue. ^c^No. D/S/total showed the number of dead (D) and sick (S) as well as the total number of chickens during the observation period. Birds that showed disease signs such as depression and ruﬄed feathers, but recovered at the end of the observation, were considered to be sick animals.*

All eight genes were sequenced to trace the origin and understand the genetic properties of the viruses. We performed phylogenetic analyses of these sequences and compared them with sequence data obtained from GenBank. BLAST^1^ analysis revealed that their HA genes were highly homologous with A/wild duck/Shandong/628/2011(H5N1) virus, and the nucleotide sequence homology was up to 97.4 and 97.6%, respectively (**Table 2**).

**Table 2:** Influenza viruses with highest nucleotide identity to each gene of 018 and 021^∗^.

Gene segment		Closest viruses in GenBank	
		Strain	Nucleotide identity, %
PB2	018	A/wild duck/Fujian/1/2011 (H5N1)	98.0%
	021		97.9%
PB1	018	A/wild duck/Fujian/1/2011 (H5N1)	97.8%
	021		97.8%
PA	018	A/wild duck/Fujian/1/2011 (H5N1)	98.4%
	021		98.4%
HA	018	A/wild duck/Shandong/628/2011 (H5N1)	97.4%
	021		97.6%
NP	018	A/hill myna/Heilongjiang/0704/2012 (H5N1)	98.3%
	021		98.0%
	018	A/wild Duck/Fujian/1/2011(H5N1)	98.0%
	021		97.7%
NA	018	A/swine/Guangdong/K6/2010 (H6N6)	98.3%
	021		98.3%
M	018	A/wild duck/Fujian/1/2011 (H5N1)	98.8%
	021		98.8%
NS	018	A/wild duck/Fujian/1/2011 (H5N1)	98.2%
	021		97.9%

*^∗^018, A/duck/Guangzhou/018/2014(H5N6); 021, A/duck/Guangzhou/021/2014(H5N6); PB, polybasic protein; PA, polymerase acidic protein; HA, hemagglutinin; NP, nucleocapsid protein; NA, neuraminidase; M, matrix protein; and NS, non-structural protein.*

**FIGURE 1:**
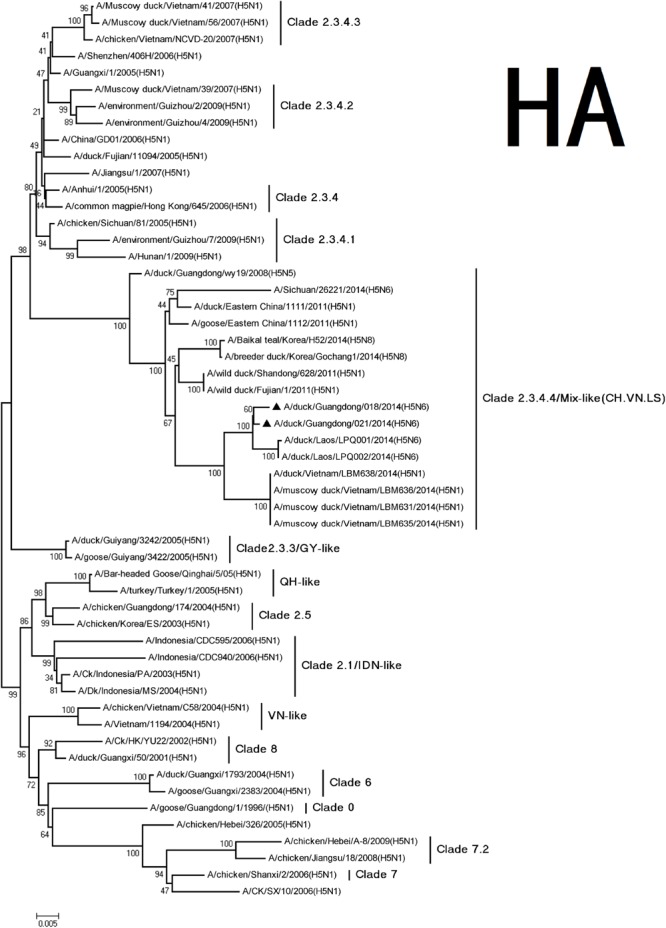
**Phylogenetic analysis of HA.** The trees were constructed using the neighbor joining method with the Maximum Composite Likelihood model and MEGA version 5.2 (www.megasoftware.net/) with 1,000 bootstrap replicates based on the following sequences: HA (A): nucleotides (nt) 29 to 1732. The triangles indicated viruses characterized in this study. Other viral sequences were downloaded from GenBank. QH, QingHai; VN, Vietnam; IDN, Indonesia; and GY, GuiYang.

According to antigenic characteristics by the World Health Organization/World Organization for Animal Health/Food, Agriculture Organization (WHO/OIE/FAO H5N1 Evolution Working Group, 2012), the HA genes of the H5N6 viruses were classified into clade 2.3.4. They could further be classified into subclade 2.3.4.4 and belong to Mix-like viruses, which include a H5N6 virus-infected human in Sichuan in 2014 (**Figure 1**) (Gu et al., 2013). They have a series of deduced basic amino acid sequences (PLRERRRKR/GLF) at cleavage sites of HA, which was characteristic of highly pathogenic AIVs (Nobusawa et al., 1991; Belser et al., 2009). Conservative amino acid residues within receptor binding pockets of HA (including E190, G225, Q226, and G228; H3 numbering) were present in the viruses. This indicated that they may retain affinity for the avian (sialic acid-2, 3-NeuAcGal) cell surface (Stevens et al., 2006). A previous study showed the S227N substitution accompanied by deglycosylation at residue 158 could substantially increase the affinity of HA for SAα2, 6Gal without reducing its binding affinity for SAα2, 3Gal (Yen et al., 2009). In our study, the H5N6 viruses simultaneously carried an S227R mutation in the receptor-binding pocket. The impact of this S227R variation with a changed residual polarity on receptor binding deserves further investigation.

**FIGURE 2:**
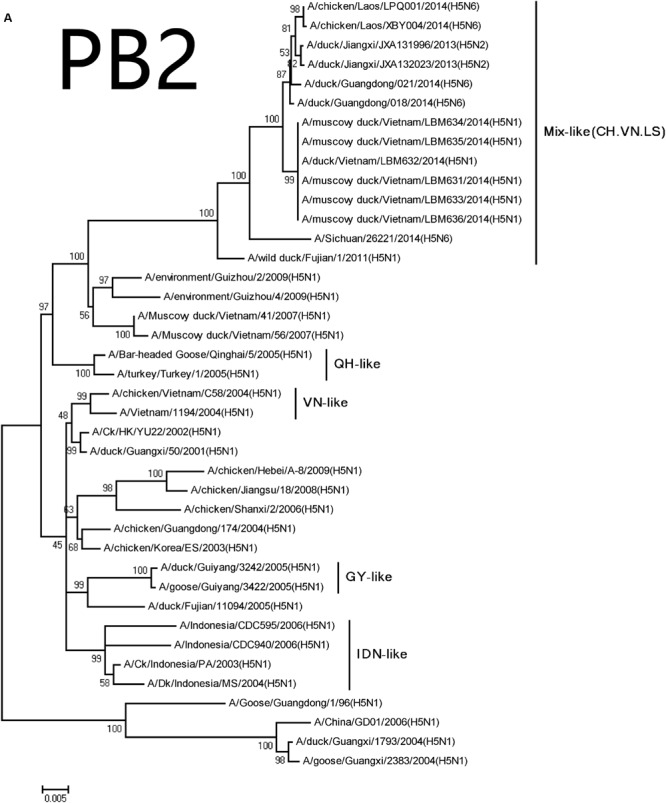

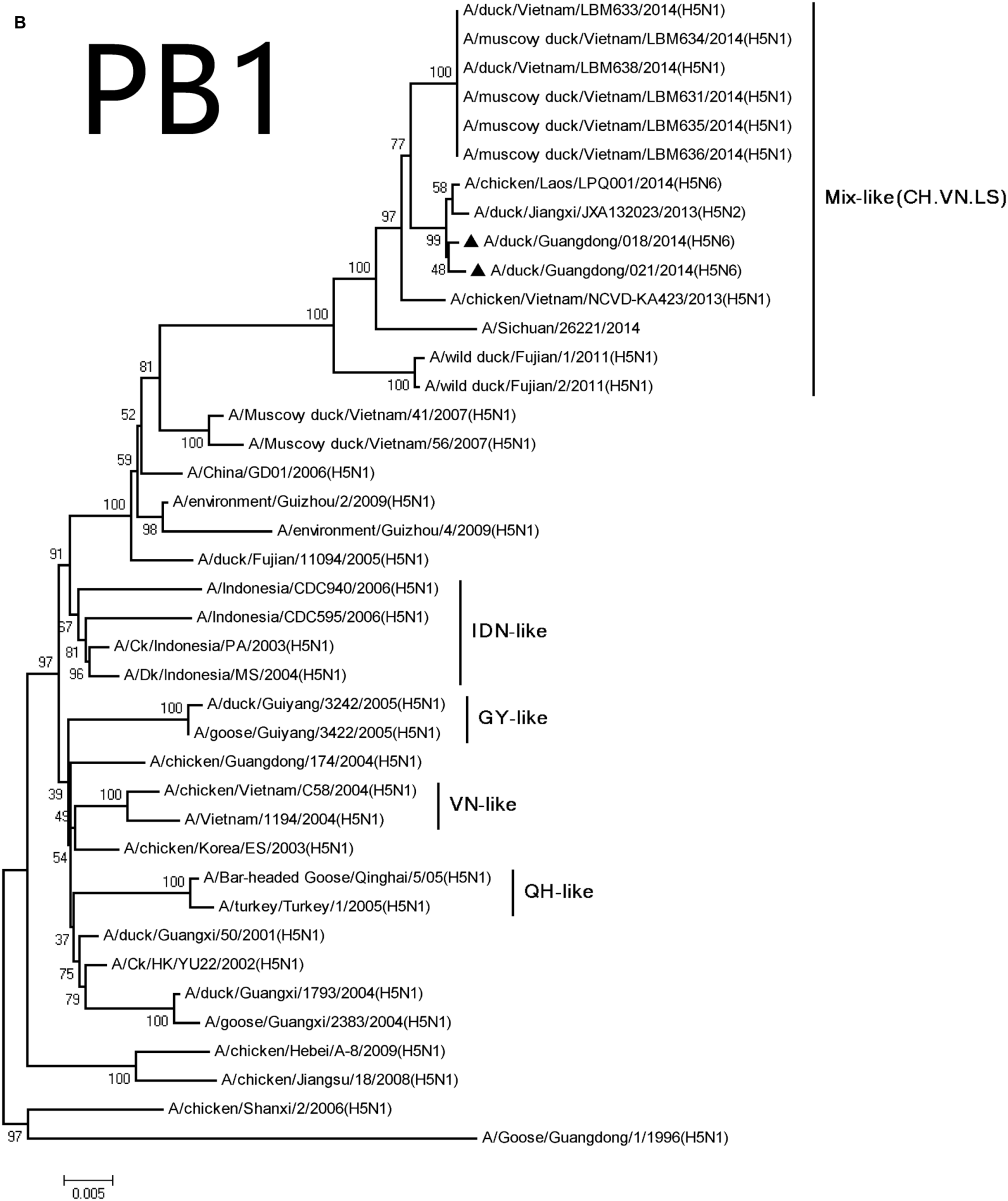

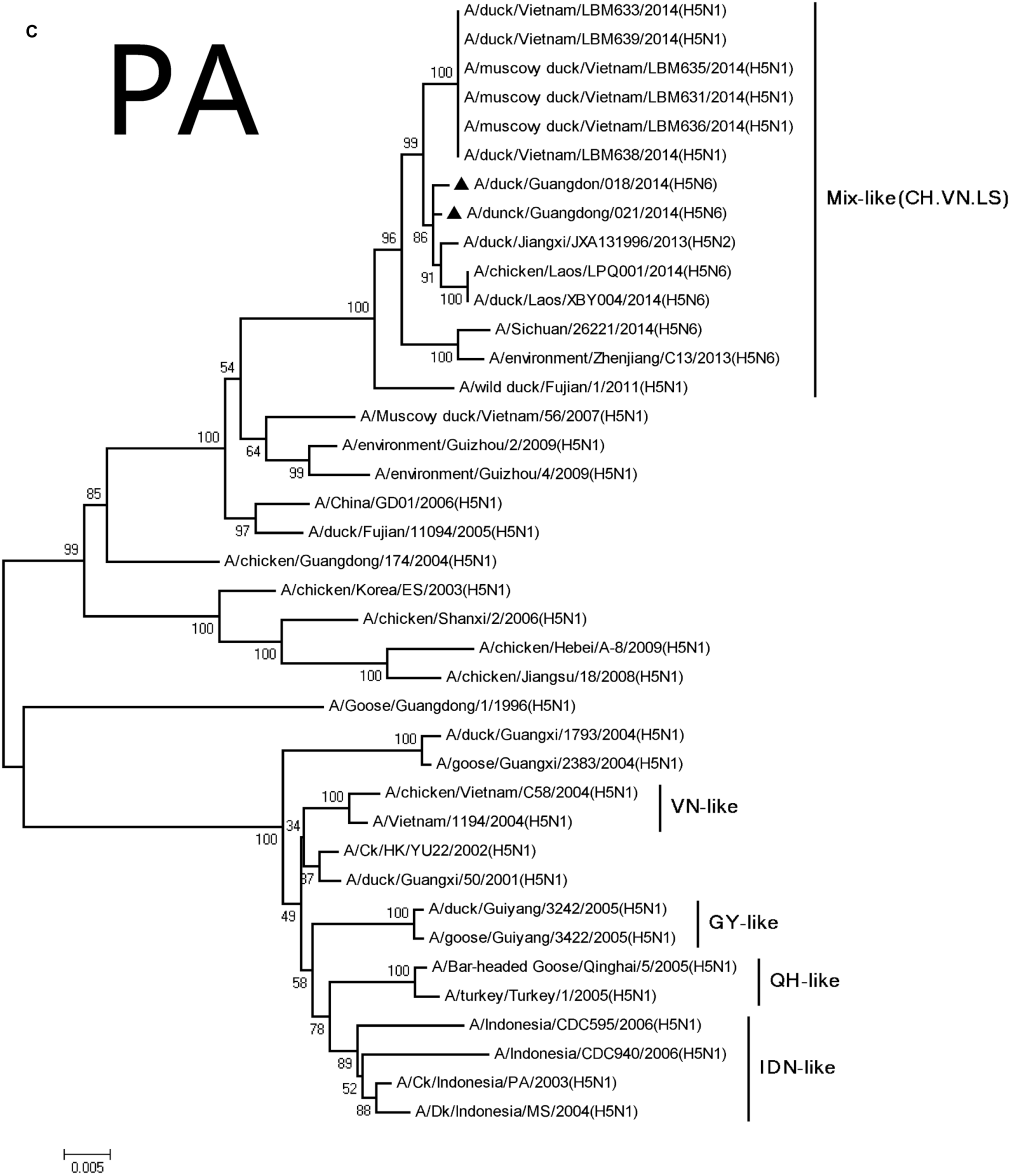

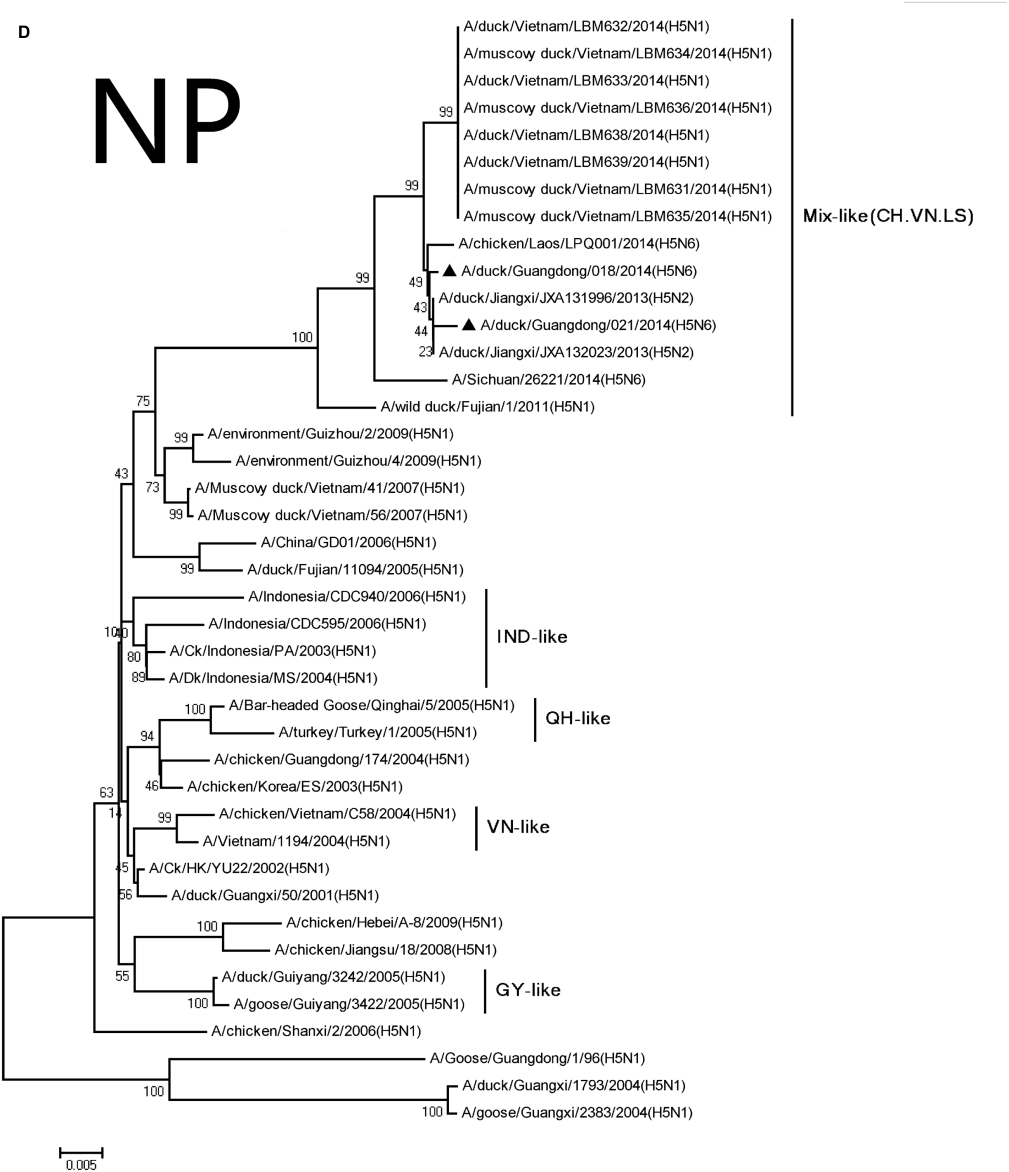

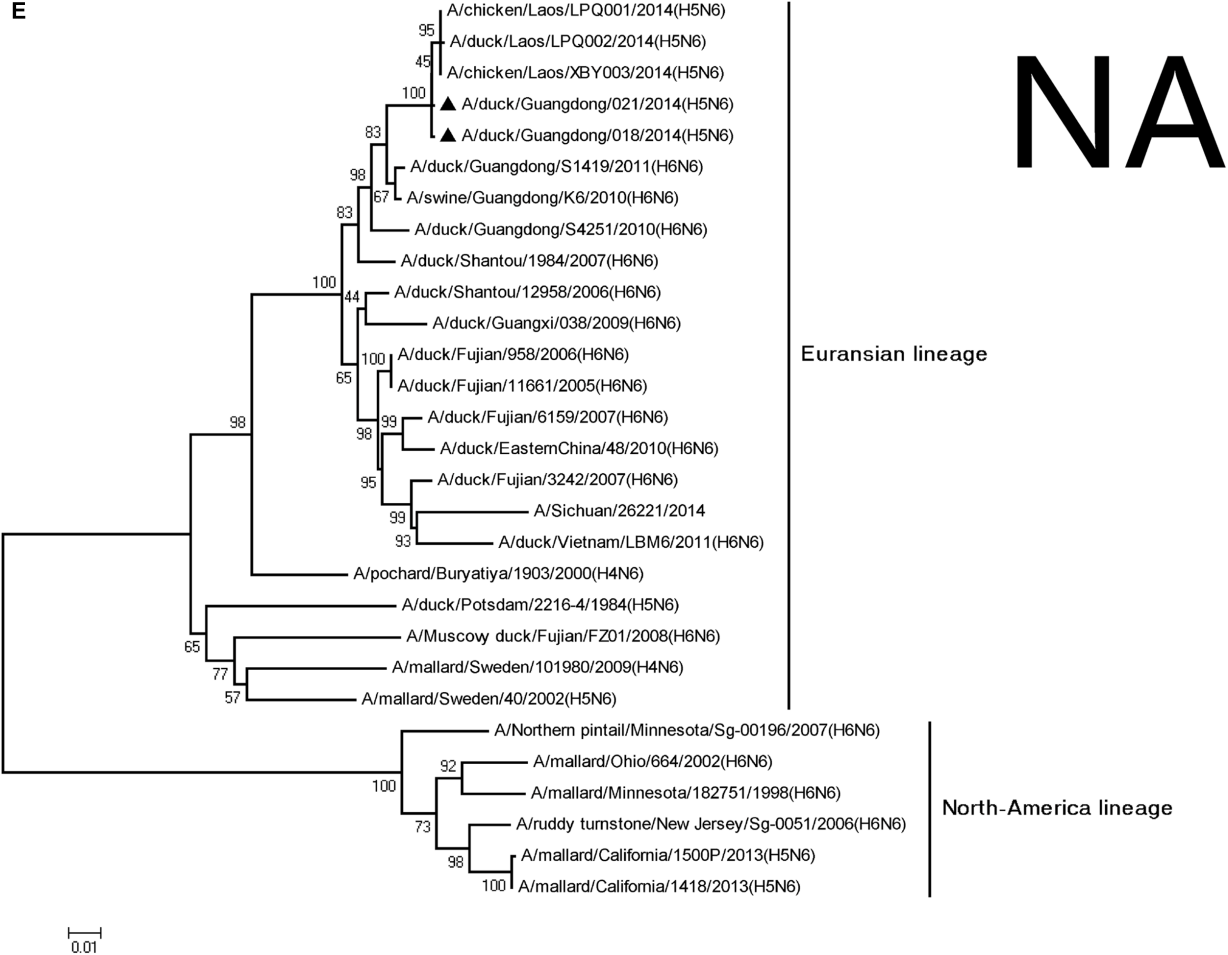

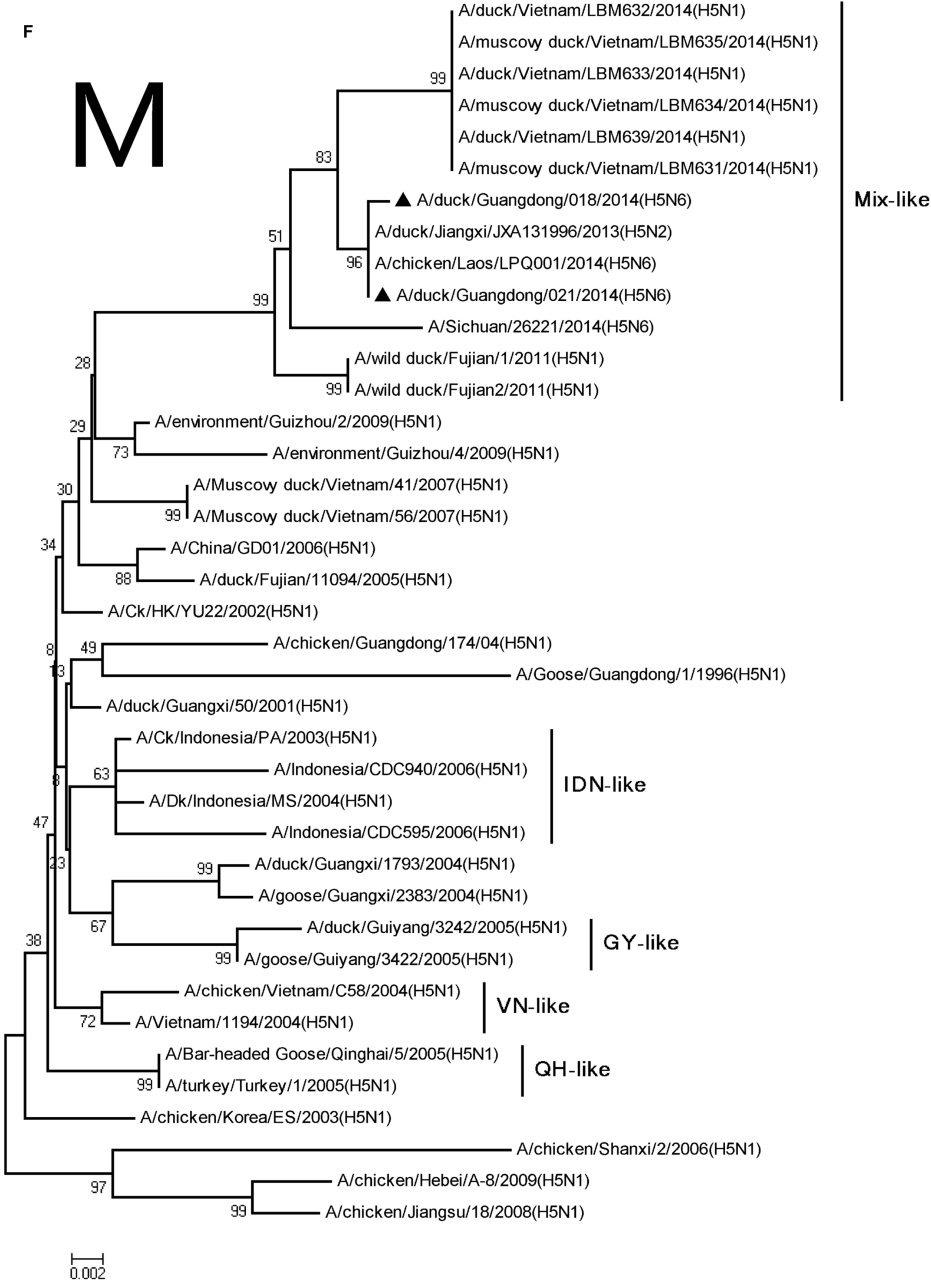

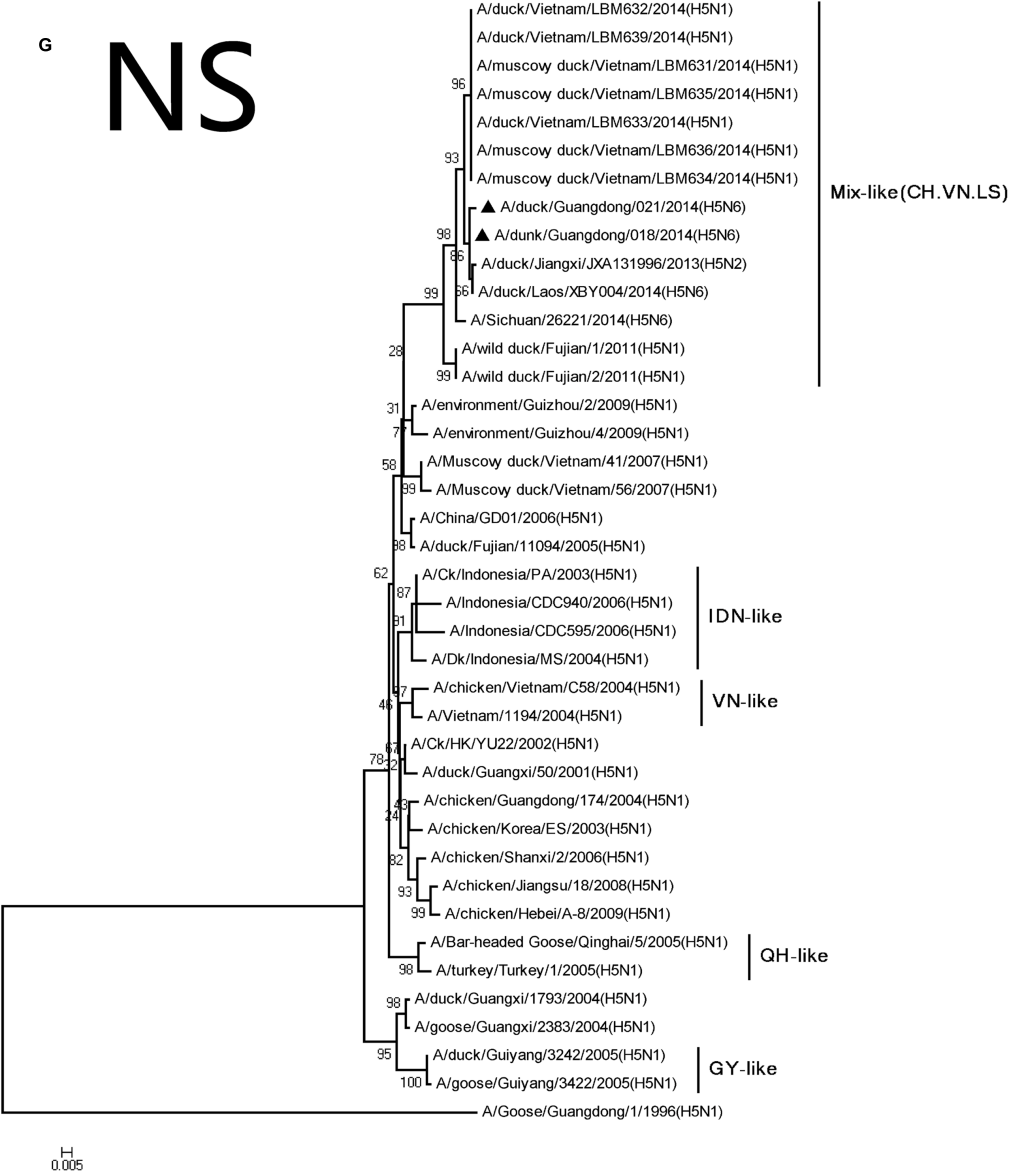
**Phylogenetic analysis of PB2 **(A)**, PB1 **(B)**, PA **(C)**, NP **(D)**, NA **(E)**, M **(F)**, and NS **(G)**.** The trees were constructed using the neighbor joining method with the Maximum Composite Likelihood model and MEGA version 5.2 (www.megasoftware.net/) with 1,000 bootstrap replicates based on the following sequences: PB2: nt 28–2307; PB1: nt 25–2298; PA: nt 25–2175; NP: nt46–1542; NA: nt19–1380; M: nt26–795; and NS: nt27–678. Except our isolate, other viral sequences were downloaded from GenBank. QH, QingHai; VN, Vietnam; IDN, Indonesia; and GY, GuiYang.

The NA genes of the viruses had high homology with A/swine/Guangdong/K6/2010 (H6N6; NA: 018, 98.3%; 021, 98.3%; **Table 2**). We retrieved complete NA sequences from GenBank (including former H5N6 viruses from North America and Europe) to compare to the viruses containing the N6 gene. The H5N6 isolates belonged to the Eurasian lineage H6N6-like viruses (**Figure 2**). An 11-amino acid deletion of residues 59–69 were found in the NA genes, but not in the previous H5N6 viruses.

The PB2 and PB1 genes were most closely related to those of the A/wild duck/Fujian/1/2011(H5N1) virus (PB2:018, 98.0%; 021, 97.9%, PB1:018, 97.8%; 021, 97.8%), respectively, (**Table 2**). The genes were clustered into Mix-like viruses, which included a H5N6 virus-infected human in Sichuan in 2014 (**Figure 2**). The PB2 627K allows avian-derived viral polymerases to evade a cellular inhibitor in human cells (Mehle and Doudna, 2008), but this mutation was not observed in PB2 of the new H5N6 viruses.

The PA and M genes nucleotide sequence homology were both 98.4 and 98.8% compared with the A/wild duck/Fujian/1/2011(H5N1) virus, respectively, (**Table 2**). The genes derived from Mix-like viruses included a H5N6 virus-infected human in Sichuan in 2014 (**Figure 2**). A S31N substitution of the M2 gene was associated with adamantane resistance, but this mutation was not observed in M2.

The NP and NS genes were highly homologous with the viruses A/hill myna/Heilongjiang/0704/2012 (H5N1) and A/wild duck/Fujian/1/2011(H5N1), respectively, (**Table 2**). The NP and NS genes were derived from the Mix-like viruses that included a H5N6-infected human in Sichuan in 2014 (**Figure 1**). The 678 amino acid residue of PB1 was S, the 615 amino acid residue of PA was K, and the 319 amino acid residue of NP was N. This indicated no genetic markers associated with increased virulence in mammals (Gabriel et al., 2005).

## Discussion

Since the late 1990s, multiple genotype H5N1 and H6N6 influenza viruses have been circulating in Chinese poultry populations (Li et al., 2004; Wang et al., 2014). This provides abundant gene pools for further inter- or intra-subtype reassortment. In recent years, an increasing number of novel avian viruses have been reported to infect humans. On May 7, 2013, the first human infection with H5N6 AIV was found in a 49-year-old man living in Nanchong City, China. This was confirmed to be the H5N6 virus from the patient’s poultry farm. Additional human infections with H5N6 were documented in December 2014. On February 9, 2015, a third human H5N6 infection was reported and was fatal.

In our study, the reassortant influenza viruses [018 (H5N6) and 021 (H5N6)] were isolated from apparently healthy domestic ducks in early March 2014. They were completely lethal in chickens (100% mortality), and the mean death time (MDT) was 0–7 DPI. The viruses could also cause horizontal transmission in chickens by naïve contact. All the naïve contact chickens in these two groups show the same clinical signs as infected chickens, and the MDT was 6 or 7 DPI. Moreover, the two viruses could replicate systemically in chickens. Virus was found in all the tested organs at high titer levels—especially the lung. The short incubation period and rapid progression of the H5N6 viruses were consistent with HPAI. Thus, our findings showed that the H5N6 viruses from waterfowl were highly pathogenic to chickens and could transmit among them.

Their HA genes were Mix-like and were assigned to clade 2.3.4.4. Their NA genes were derived from H6N6-like Eurasian lineage viruses; the internal genes included M, NS, NP, PA, PB1, and PB2 derived from Mix-like viruses. In addition, the H5N6 virus-infected human in Sichuan in 2014 also belongs to the Mix-like viruses. Such Mix-like gene pools are characterized by H5N1 and H5N6 subtypes of AIVs isolated from Vietnam and Laos. In addition, a small amount of H5N1 and H5N2 viruses were isolated in China. The H5N1 gene pool has widened and emerged since 2003.

**FIGURE 3:**
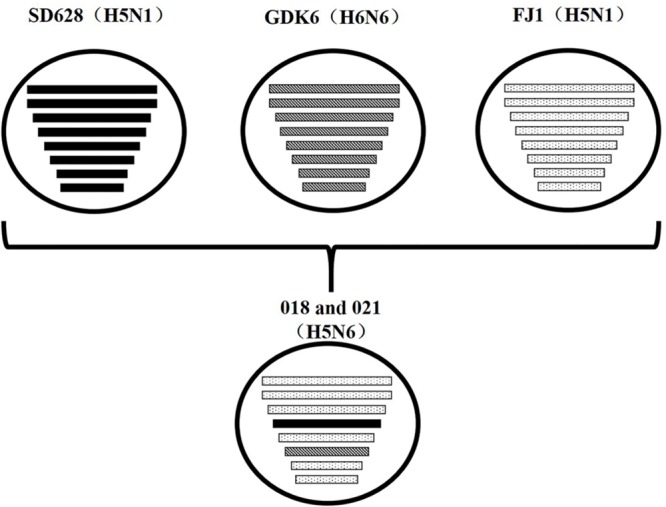
**Putative genomic compositions of novel H5N6 influenza viruses isolated from domestic ducks in Southern China 2014 as well as their five possible parent viruses.** The eight gene segments (from top to bottom) in each virus were basic polymerase 2, basic polymerase 1, acidic polymerase, hemagglutinin, nucleoprotein, neuraminidase, matrix, and non-structural protein. Each pattern represented a separate virus background. Blank indicated SD628 (H5N1); A/wild duck/Shandong/628/2011 (H5N1); bias indicated GDK6 (H6N6), A/swine/Guangdong/K6/2010(H6N6); spot indicated FJ1 (H5N1), A/Wild duck/Fujian/1/2011(H5N1). The simplified schematic illustration was based on nucleotide-distance comparison and phylogenetic analysis.

In the future, the community must study whether this Mix-like gene pool will become more diverse and even branch into a new subtype/genotype. Moreover, the H5N6 avian influenza broke out in Laos in late March 2014. The highly homologous nucleotide in our study suggested that our isolates might have an identical origin with the A/duck/Laos/LPQ001/2014(H5N6) virus from Laos. The NA genes of our isolates and the LPQ001 virus were highly homologous with the virus A/swine/Guangdong/K6/2010(H6N6). The other genes of our viruses and the LPQ001 virus were highly homologous with the H5N1 viruses from Fujian, respectively, (**Figure 3**). Moreover, the data suggested that the HA genes and the internal genes of H5N1 viruses from Vietnam are highly related to those from the H5N6 viruses. Therefore, our results suggested that the H5N6 viruses might circulate among Southern China, Vietnam and Laos.

Reassortant among different influenza viruses is considered the main mechanism for the emergence of new viruses. The co-circulation of H5 and H6 influenza viruses in domestic ducks contributed to the antigenic and genetic diversity of influenza viruses in Southern China. Zhejiang, Hubei, and Guangdong in particular had an outbreak of H5N6 avian influenza in farms in 2014 followed by Hunan and Jiangsu in 2015 (World Organization for Animal Health, 2015). In addition, the viruses were reported to have infected humans in Sichuan, Guangdong, and Yunnan, China from 2014 to the present. As these viruses caused 100% mortality in chickens, these viruses should be monitored in poultry. Their risk to human health cannot be denied and should be monitored in future.

## Author Contributions

PJ and JC designed this study, performed the experiments and participated in the data collection and analysis. YS, HS, ZZ, QY, SW, NQ, NW, and GO assisted with animal experiment. PJ and JC drafted the manuscript. ML participated in writing the discussion.

## Conflict of Interest Statement

The authors declare that the research was conducted in the absence of any commercial or financial relationships that could be construed as a potential conflict of interest.

## Footnotes

1 www.ncbi.nlm.nih.gov/genomes/FLU/FLU.html
